# Polyphosphazene-Based Anion-Anchored Polymer Electrolytes For All-Solid-State Lithium Metal Batteries

**DOI:** 10.1021/acsomega.3c10311

**Published:** 2024-03-19

**Authors:** Billy R. Johnson, Ashwin Sankara Raman, Aashray Narla, Samik Jhulki, Lihua Chen, Seth R. Marder, Rampi Ramprasad, Kostia Turcheniuk, Gleb Yushin

**Affiliations:** † School of Materials Science and Engineering, Georgia Institute of Technology, Atlanta, Georgia 30332, United States; ‡ School of Chemistry and Biochemistry, Georgia Institute of Technology, Atlanta, Georgia 30332, United States

## Abstract

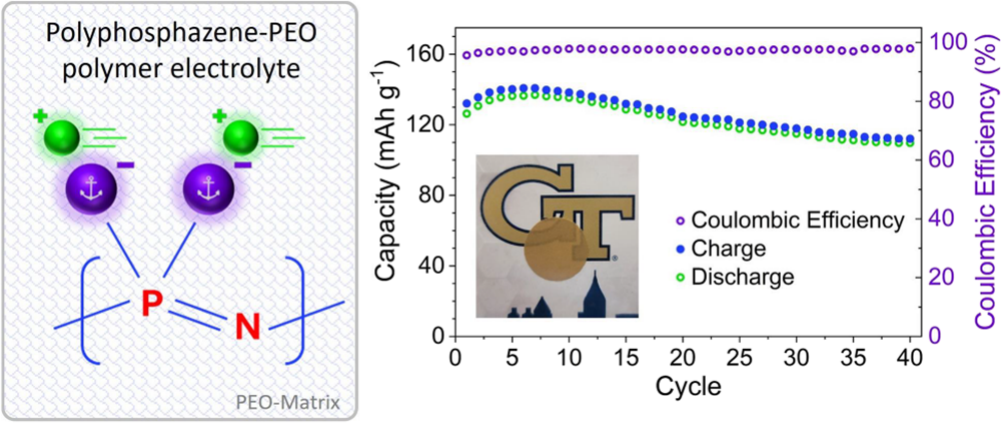

Safety concerns of traditional liquid electrolytes, especially when paired with lithium (Li) metal anodes, have stimulated research of solid polymer electrolytes (SPEs) to exploit the superior thermal and mechanical properties of polymers. Polyphosphazenes are primarily known for their use as flame retardant materials and have demonstrated high Li-ion conductivity owing to their highly flexible P = N backbone which promotes Li-ion conduction via inter- and intrachain hopping along the polymer backbone. While polyphosphazenes are largely unexplored as SPEs in the literature, a few existing examples showed promising ionic conductivity. By anchoring the anion to the polymer backbone, one may primarily allow the movement of Li ions, alleviating the detrimental effects of polarization that are common in conventional dual-ion conducting SPEs. Anion-anchored SPEs, known as single Li-ion conducting solid polymer electrolytes (SLiC-SPEs), exhibit high Li-ion transference numbers (*t*_Li^+^_), which limits Li dendrite growth, thus further increasing the safety of SPEs. However, previously reported SLiC-SPEs suffer from inadequate ionic conductivity, small electrochemical stability windows (ESWs), and limited cycling stability. Herein, we report three polyphosphazene-based SLiC-SPEs comprising lithiated polyphosphazenes. The SLiC polyphosphazenes were prepared through a facile synthesis route, opening the door for enhanced tunability of polymer properties via facile macromolecular nucleophilic substitution and subsequent lithiation. State-of-the-art characterization techniques, such as differential scanning calorimetry (DSC), electrochemical impedance spectroscopy (EIS), and solid-state nuclear magnetic resonance spectroscopy (ssNMR) were employed to probe the effect of the polymer structure on Li-ion dynamics and other electrochemical properties. Produced SPEs showed thermal stability up to ∼208 °C with ionic conductivities comparable to that of the best-reported SLiC-SPEs that definitively comprise no solvents or plasticizers. Among the three lithiated polyphosphazenes, the SPE containing dilithium poly[bis(trifluoroethylamino)phosphazene] (pTFAP2Li) exhibited the most promising electrochemical characteristics with *t*_Li^+^_ of 0.76 and compatibility with both Li metal anodes and LiFePO_4_ (LFP) cathodes; through 40 cycles at 100 °C, the PEO-pTFAP2Li blend showed 81.2% capacity utilization and 86.8% capacity retention. This work constitutes one of the first successful demonstrations of the cycling performance of a true all-solid-state Li-metal battery using SLiC polyphosphazene SPEs.

## Introduction

The landscape of energy storage continues to expand with the growing adoption of electric vehicles (EVs) and portable consumer electronics.^1^ The predicted climate change further requires rapid transitioning into grid-based storage of energy harnessed from renewable sources.^2^ Lithium-ion batteries (LIBs) continue to play a key role in this, but safety concerns of liquid electrolyte-based LIBs have somewhat limited their applications.^3^ These safety concerns are related to the high vapor pressure in combination with the inherent flammability of most organic liquid electrolytes and typically originate from a thermal runaway reaction often caused by internal short-circuits induced, for example, by Li dendrite formation that can pierce the separator or externally induced LIB damages.^4,5^ These issues become significant when Li metal is used as an anode (as in a Li-metal battery, LMB), which offers high theoretical specific capacity (3860 mAh g^–1^), low density (0.59 g cm^–3^), the lowest standard potential (−3.04 V vs. SHE), and thus very high cell-level energy density.^6,7^ Recent studies demonstrated that the formation of a stable solid electrolyte interphase (SEI) layer may minimize the probability of thermal runaway reactions.^8,9^ However, such SEIs are often brittle and prone to damage under abuse, which may limit the viability of liquid electrolytes in practical applications of LMBs. Thus, many researchers believe that solid electrolytes may become better alternatives, as they are thermally more stable, mechanically more robust, may suppress the Li dendrite growth, are less flammable, and thus may prevent catastrophic battery failures.^10−12^

All-solid-state LMBs have their own set of challenges. First, the very solid nature of the electrolyte may make the complete “wetting” of the porous electrodes difficult, thus creating voids, which may increase the resistance of the cells and lower their capacity utilization and volumetric energy density. Second, Li dendrites may grow more easily through such voids and cause LMB cells to short. Third, solid electrolytes may react with the Li metal, causing undesirable side products, which may lower Coulombic efficiency and ultimately cause gradual/rapid cell failure. Addressing these challenges using solid polymer electrolytes (SPEs) has been the subject of extensive research in the last few decades. Most research focused on PEO/lithium bis(trifluoromethanesulfonyl)imide (LiTFSI) systems and has now expanded into a variety of polymer hosts and lithium salts.^13−18^

Polyphosphazenes, a class of polymers with an alternating P = N backbone, are a promising alternative to PEO-based SPEs. The highly flexible backbone gives rise to extremely low glass transition temperatures, and this high degree of segmental mobility is a major driver in Li-ion conduction. They exhibit superior flame-retardant properties by promoting the formation of char residue when heated to extreme temperatures and by releasing ammonia, nitrogen, and water which dilute flammable gases and absorb heat which further enhances the safety of SPEs.^19^ Polyphosphazene chemistry has existed for decades and offers a unique synthesis advantage over other polymers through simple macromolecular nucleophilic substitution of P–Cl bonds in a reactive precursor polymer (poly(dichlorophosphazene); PDCP).^20^ Substitution of the polyphosphazene backbone by alkoxy and aryloxy substituents has been explored in LIBs aiming to achieve greater ionic conductivity in SPEs with flame retardant properties.^20,21^ For example, Blonsky et al. introduced the first polyphosphazene-based SPE, poly[bis(2-(2-methoxyethoxy)ethoxy)phosphazene] (MEEP) and demonstrated higher conductivity than in PEO-Li salt systems.^22,23^ However, MEEP is a gum-like polymer, which presents poor dimensional stability, and the high ionic conductivity was shown to be a result of significant anion transport with low *t*_Li^+^_.^24,25^ These early examples in the literature mostly focus on oligoether substituents with structures similar to that of PEO and contribute high conductivity values (up to 10^–4^ S cm^–1^ at 30 °C) with good thermal and electrochemical stability with lithium metal anodes; however, low *t*_Li^+^_ suggests that the majority of this conductivity can be attributed to the anionic movement, which limits the performance of these types of SPEs. In such SPEs, both the cation and anion are mobile and are referred to as dual-ion conducting SPEs. During cycling, cations and a higher fraction of electrochemically inactive anions move in opposite directions resulting in a low transference number (generally *t*_Li^+^_ < 0.5; often < 0.2) creating significant concentration gradients and cell polarization, which gives way to undesirable side reactions and premature cell failure.^24,25^ This has led to the development of polymers, with anionic groups covalently attached to the polymer backbone resulting in transference numbers greater than 0.5, known as single Li-ion conductors (SLiCs).^26,27^ Immobilizing the anionic species not only reduces polarization but also has been proven to suppress dendrite growth and thus significantly improve cell performance and lifetimes.^28,29^ Furthermore, computations show comparable performance in electrolytes with *t*_Li^+^_ approaching unity to that of conventional dual-ion SPEs with conductivities an order of magnitude higher.^30^ A large variety of SLiC-SPEs have been developed in the past decade based on a carbon backbone with efforts to fine-tune mechanical and electronic properties through a variety of challenging and expensive synthetic methods resulting in only moderate improvements in electrochemical performance. Alternatively, the facile synthesis of polyphosphazenes allows one to easily synthesize SLiC polyphosphazenes with the anion covalently attached to the polymer backbone. Anchoring the anion to the polymer backbone to produce SLiC polyphosphazenes provides a potential route to overcome the low *t*_Li^+^_ of MEEP salt-in-polymer systems; however, only a few relevant examples exist in the literature.^31−33^ In fact, we are not aware of any polyphosphazene-based SLiC-SPEs that have been used as an electrolyte in a solid-state battery that could be cycled. Thus, we revisited the polyphosphazene chemistry to create SLiC polyphosphazenes and applied them to the formation of fully functional cells in this work.

**Scheme 1:**
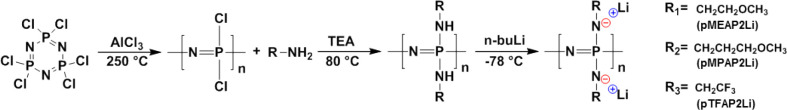
Synthesis Route to Lithiated Polyphosphazenes

Herein, we report three novel polyphosphazene-based SLiC polymers, namely, dilithium poly[bis(methoxyethylamino)phosphazene] (pMEAP2Li), dilithium poly[bis(methoxypropylamino)phosphazene] (pMPAP2Li), and dilithium poly[bis(trifluoroethylamino)phosphazene] (pTFAP2Li) (Scheme 1) and investigate their physiochemical properties by experimental and computational methods. This work establishes a facile synthesis route to single-ion conducting polymers based on simple macromolecular substitution of a polyphosphazene parent polymer, providing the blueprint for new SLiC-SPEs. The facile synthesis can be further extended to mixed-substituent derivatives with two or more distinct substituents for enhanced tunability of the polymer’s properties. Through a thorough optimization process, we demonstrate, for the first time for a polyphosphazene-based SLiC-SPE, the cycling performance of *all-solid-state* LMBs.

## Results and Discussion

### Synthesis and Characterization

All three lithiated polyphosphazenes are prepared according to Scheme 1, each beginning with the synthesis of the reactive precursor polymer, PDCP, via thermal ring-opening polymerization of hexachlorocyclotriphosphazene (HCCP) in the presence of catalytic amounts of AlCl_3_ (see the Experimental Section for details). The chlorine atoms of PDCP are then replaced with stoichiometric amounts of a primary amine to obtain alkylamido-substituted polyphosphazenes. The “NH” protons of the “P-NH-R” moiety are subsequently replaced with Li-ions by reacting with *n*-butyllithium (*n*-buLi) to form the SLiC polyphosphazene. The synthesized SLiC polyphosphazenes are characterized via ^1^H, ^13^C, ^31^P, and ^7^Li solution NMR spectroscopies to confirm their chemical structures and purities (Figure S1). The disappearance of the proton peak on the amine group, combined with the presence of a ^7^Li signal, suggests complete or near-complete lithiation. Furthermore, characteristic peak broadening is observed in the ^1^H, ^13^C, and ^31^P spectra for the lithiated polyphosphazenes, which suggests the presence of the magnetically active Li nuclei in the polyphosphazene backbone and successful lithiation.

### Computational Evaluation of ESW

**Figure 1:**
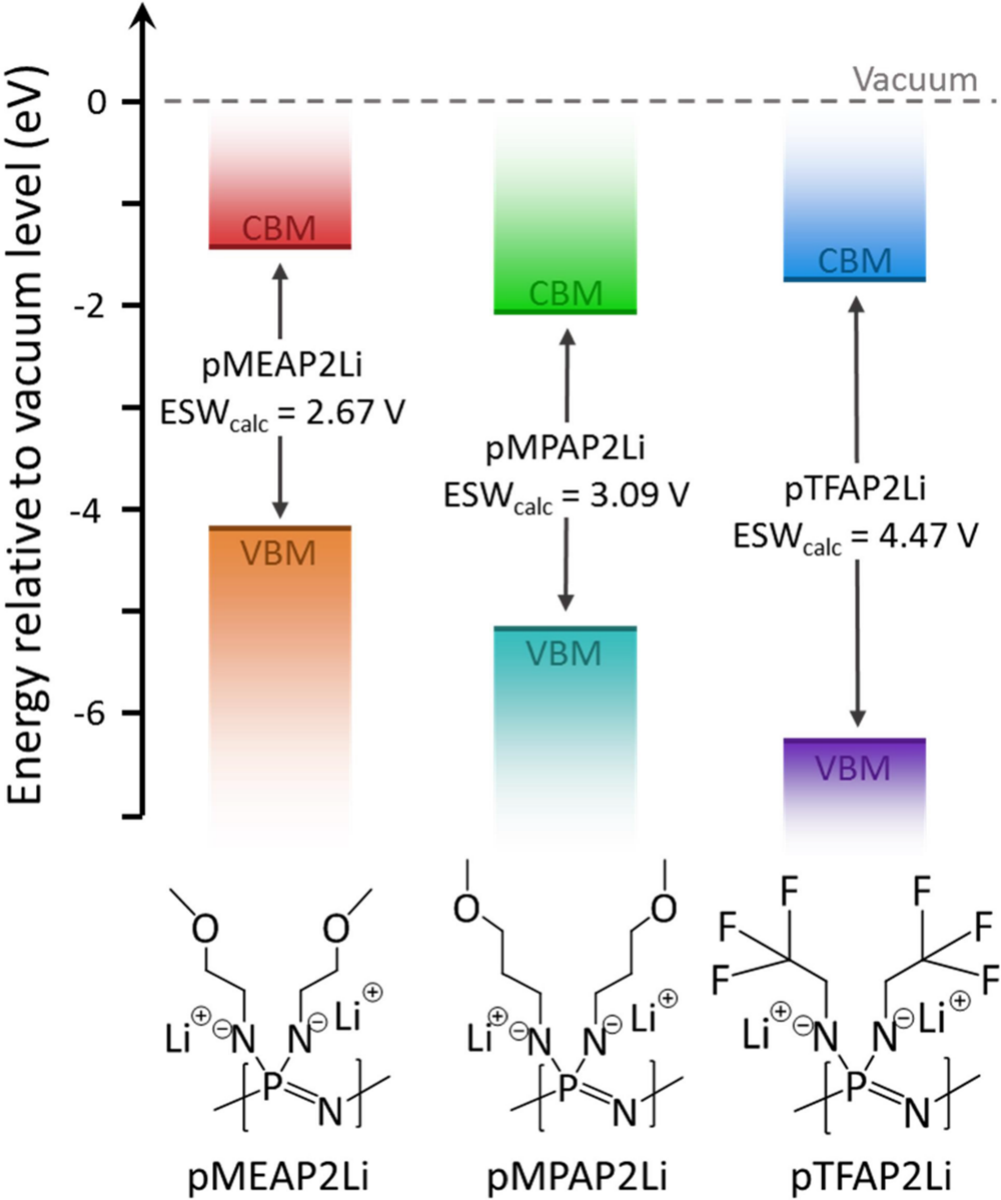
Energy diagram showing the electrolyte interface with the anode (CBM) and the cathode (VBM) for each of the three lithiated polyphosphazenes. The difference between the CBM and the VBM is the ES*W*_calc_.

Density functional theory (DFT) was employed to determine the polymer with the widest electrochemical stability window (ESW) for use with higher-voltage cathode materials. In simpler terms, the ESW is determined by the reduction and oxidation potential of the SPE, which can be modeled as the conduction band minimum (CBM) and the valence band maximum (VBM), respectively.^34^Figure 1 shows these modeling results. Based on these calculations, pTFAP2Li is the only one of the three that meets the voltage requirements for the most used Li-ion cathode materials (LiCoO_2_ (LCO), LiMn_2_O_4_ (LMO), LiFePO_4_ (LFP), and LiNi_*x*_Mn_*y*_Co_*z*_O_2_ (*x* + *y* + *z* = 1; NCM)).

### Physical and Electrochemical Characterization of SLiC Polymer Electrolytes

**Figure 2:**
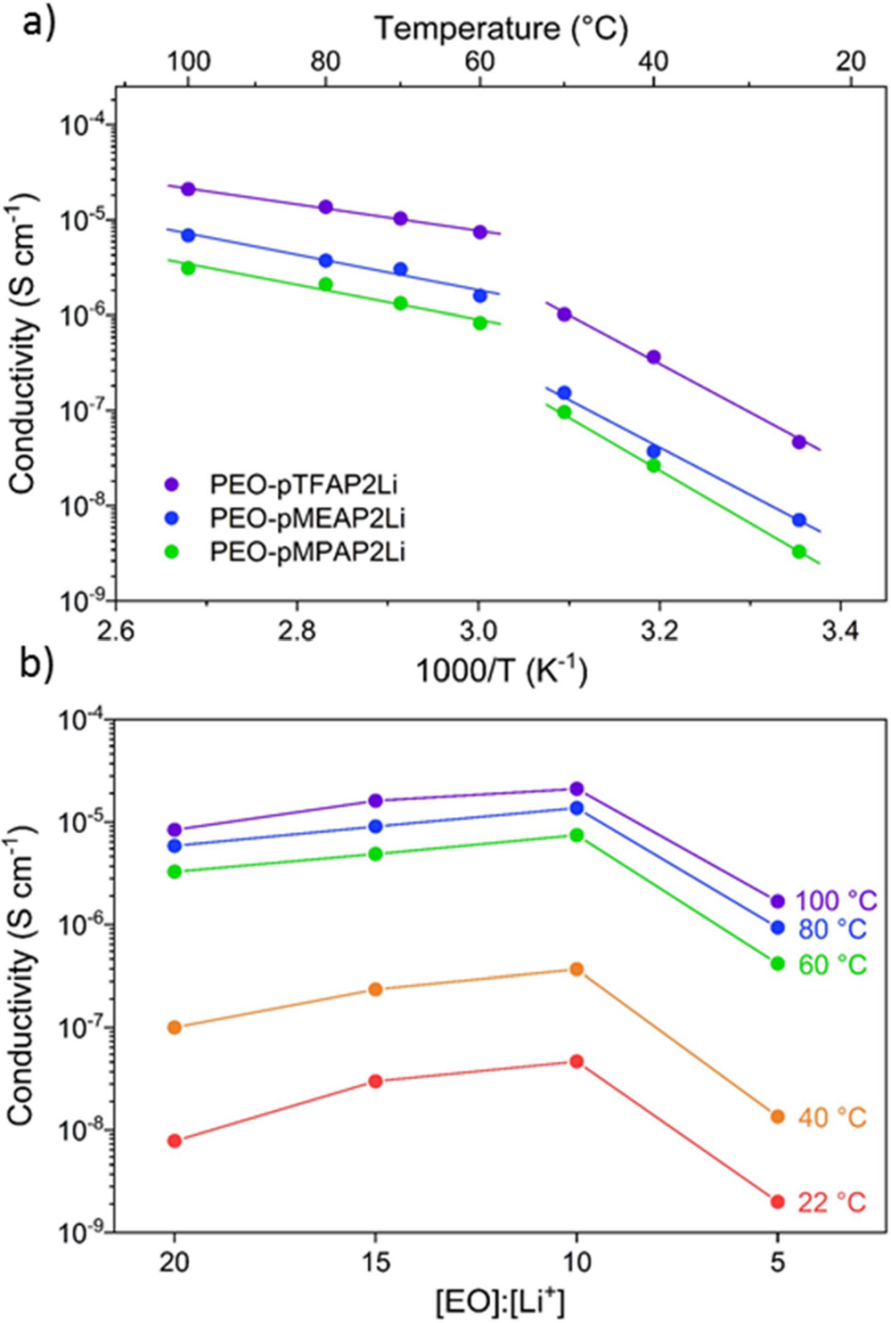
(a) Temperature dependence of ionic conductivity in all the lithiated polyphosphazene blended polymer electrolytes ([EO]:[Li^+^] = 10:1 for all) with linear fits corresponding to amorphous PEO regions above 60 °C and semicrystalline PEO below 60 °C. (b) Ionic conductivities of PEO-pTFAP2Li with different [EO]:[Li^+^] ratios.

Due to the powdery state of the lithiated polyphosphazenes, they were blended with PEO to improve the mechanical properties of the SLiC-SPEs. Free-standing polymer electrolyte membranes were prepared by casting blended solutions of lithiated polyphosphazenes with PEO at a 10:1 [EO]:[Li^+^] ratio for comparison (EO: one ethylene oxide repeat unit of PEO and two Li-ions per lithiated polyphosphazene repeat unit). These films had a controllable thickness of ∼75 μm for this study. It should be noted that there was no plasticizer or added Li salt used in these studies. The ionic conductivity of the PEO-blended polyphosphazenes was determined by electrochemical impedance spectroscopy (EIS) in stainless steel symmetric cells (Figure 2a). All blended electrolytes exhibit relatively low room temperature ionic conductivity (ca. 3 × 10^–9^–5 × 10^–8^ S cm^–1^) but show a significant increase in conductivity around 60 °C when PEO transitions from a semicrystalline phase to an amorphous phase and enters a soft-state of viscous flow.^35−37^ As such, one observes two linear regimes of ionic conductivity dependence on temperature. The temperature-dependent ionic conductivity of the two regions (semicrystalline and amorphous) can be separately defined by the Arrhenius equation:
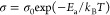
1where σ is the ionic conductivity, σ_0_ is a pre-exponential factor, *E*_a_ is the activation energy, *k*_B_ is the Boltzmann constant and *T* is the absolute temperature. The significantly greater activation energies (e.g., 1.0–1.1 eV at room temperature for this work; for comparison, other PEO/LiTFSI systems report activation energies around 0.5 eV at room temperature^38,39^) at lower temperatures demonstrate that all Li-ions are moderately/tightly ion-paired with the negatively charged nitrogen centers of lithiated polyphosphazenes compared to highly dissociated salts such as LiTFSI. This suggests that the effect of PEO in minimizing the ion-pairing is marginal at lower temperatures when it is semicrystalline, which limits the lower-temperature application of such produced SPEs. At higher temperatures (≥60 °C), the activation energies are considerably reduced (i.e., ionic conductivity increases), as thermal energies should minimize ion-pairing effects of the lithiated polymer, which should be further promoted by phase transition of PEO that aids Li-ion conduction by increased segmental motion.^36^ Comparing the three lithiated polyphosphazene blends (Figure 2a and Table S1), the PEO-pTFAP2Li system shows the highest ionic conductivity (2.1 × 10^–5^ S cm^–1^ at 100 °C) and the lowest activation energy (*E*_a_ = 0.28 eV). This is likely due to the inductive effect of the electron-withdrawing CF_3_ groups that weaken the ion pairing between the Li-ion and negatively charged nitrogen atom compared to other synthesized SLICs. Having the highest ionic conductivity and the highest calculated oxidation potential (VBM), the PEO-pTFAP2Li blended SLiC-SPE was chosen for further analysis in this work.

To evaluate the dependence of conductivity upon [EO]:[Li^+^], the ratio was varied from 5:1 up to 20:1 for the PEO-pTFAP2Li SLIC-SPE (Figure 2b and Table S2). As expected, increasing the pTFAP2Li content, thus increasing the number of charge carriers in the SLIC-SPE, resulted in a marginal increase in conductivity; however, a limit was reached in the 5:1 SLIC-SPE where the ionic conductivity decreased significantly. This is likely a result of reduced Li-ion mobility as dissociated ions transiently act as cross-links between polymer chain segments and ultimately slow intrachain mobility at excessive charge carrier concentrations.^40,41^

**Figure 3:**
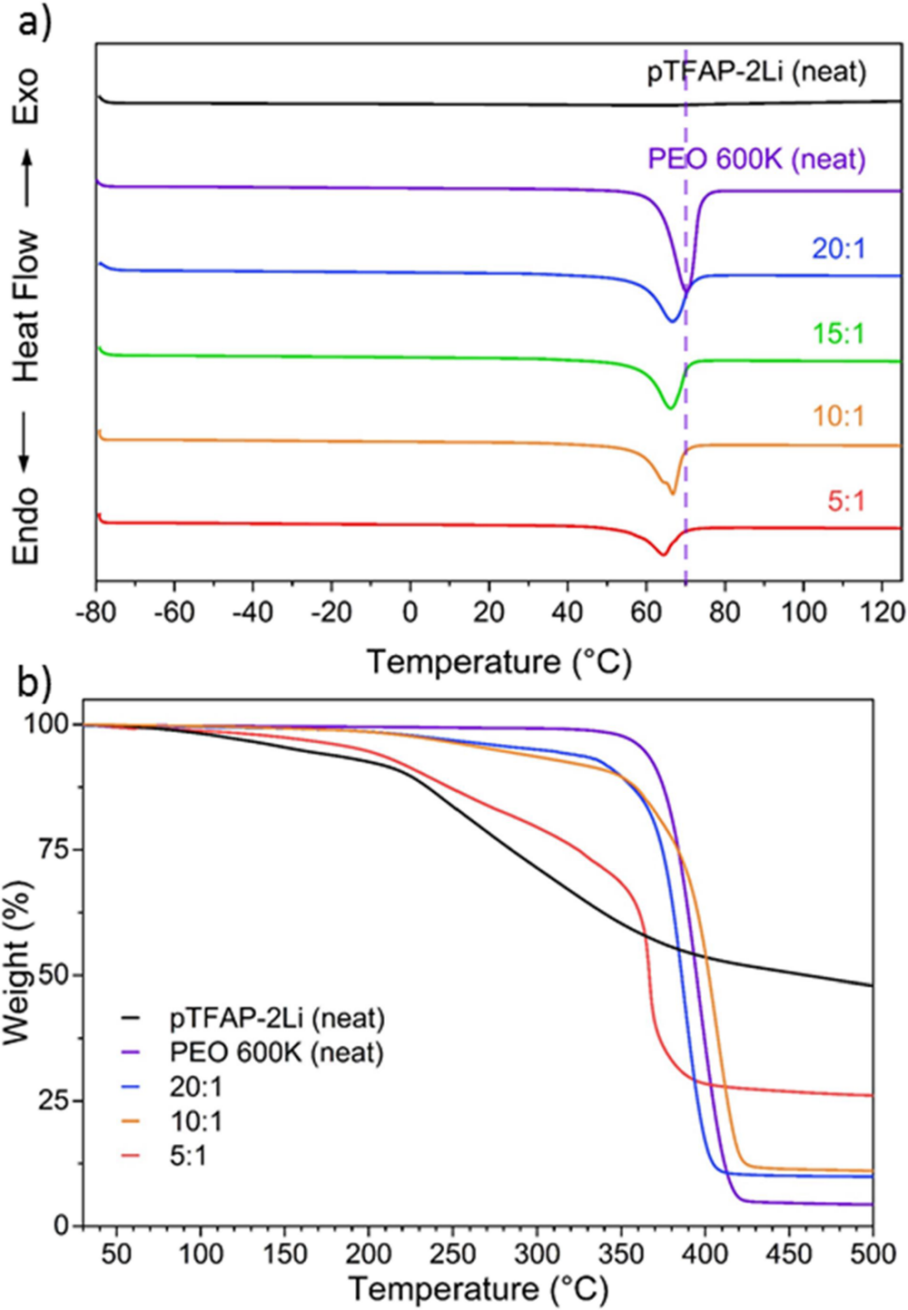
(a) DSC and (b) TGA traces of PEO-pTFAP2Li SPEs at various [EO]:[Li^+^] ratios.

Thermal properties of the PEO-pTFAP2Li electrolyte at various [EO]:[Li^+^] ratios were evaluated with differential scanning calorimetry (DSC) and thermogravimetric analysis (TGA) (Figure 3a,b, respectively). DSC revealed no other discernible phase transitions, except the expected melting behavior of PEO. PEO melting temperature decreased with increasing pTFAP2Li content, and it can be inferred that pTFAP2Li has a plasticizing effect on the PEO polymer matrix, stabilizing the amorphous phase. This is consistent with the observed crystallinity trend, with the 5:1 blend being the least crystalline (Table 1^42^).

**Table 1:** Phase Transition Behavior of SLiC-SPEs at Various [EO]:[Li^+^] Ratios

sample	EO:Li^+^	*T*_m_ (°C)^a^	Δ*H*_m_ (J g^–1^)^b^	crystallinity (%)^c^
PEO600 K (neat)		71.1	171.02	84.2
PEO-pTFAP2Li	20:1	65.3	103.39	50.9
	15:1	65.3	93.77	46.2
	10:1	64.9	86.36	42.5
	5:1	64.5	56.43	27.8
pTFAP2Li (neat)				

a Melting temperature.

b Enthalpy of melting.

c Crystallinity of the blended SLiC-SPEs calculated by Δ*H*_m_/Δ*H*_0_, where Δ*H*_m_ is the specific enthalpy of PEO and Δ*H*_0_ is 203 J g^–1^, a literature value for the specific enthalpy of a 100% crystalline PEO.^42^

This is also consistent with the conductivity data as crystalline regions act as ionic insulators and polymers that are more amorphous display higher ionic conductivity. TGA was used to probe the thermal stability of PEO-pTFAP2Li blends compared with the neat forms of the two constituents in the blend, as shown in Figure 3b. The decomposition of the lithiated polyphosphazene limits the thermal stability to 208 °C for the blended SPE, which is sufficient for most LIB applications. Flame tests conducted for neat pTFAP2Li pellet show excellent flame resistance under a propane torch for durations as long as 20 s and initial combustion in the PEO-pTFAP2Li blend can be attributed to the properties of the host polymer PEO as shown in Figure S2.

**Figure 4:**
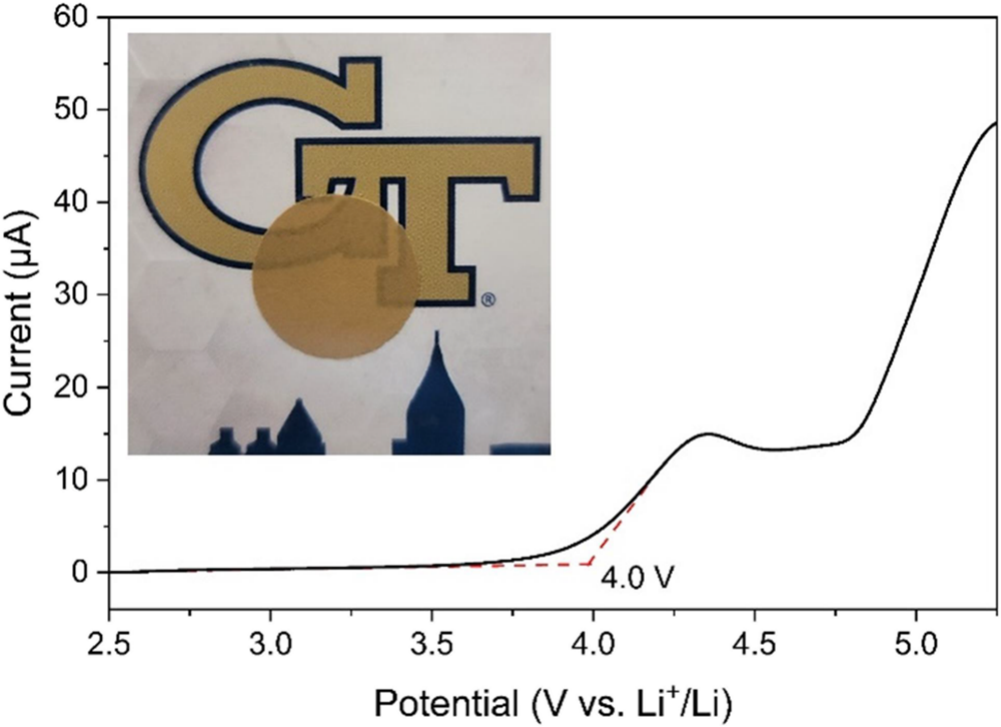
Linear sweep voltammogram of 10:1 PEO-pTFAP2Li SLiC-SPE from the OCV to 5.5 V at 0.2 mV s^–1^ (inset: optical image of the PEO-pTFAP2Li membrane).

Next, we examined the ESW of the 10:1 PEO-pTFAP2Li blend via linear sweep voltammetry in an asymmetric cell with stainless steel as the working electrode and Li metal as the reference/counter electrode with a scan rate of 0.2 mV s^–1^. The blend was found to be oxidatively stable up to about 3.7–4.0 V, after which the PEO matrix begins to oxidize (Figure 4).^18,43^ The second oxidation event at 4.7 V is likely a result of the oxidative decomposition of pTFAP2Li, which is in relatively good agreement with the computational evaluations for the VBM (oxidation potential) of pTFAP2Li. In total, it is established that any cell operation may need to be performed below 4.0 V (vs. Li/Li^+^) to ensure the stability of the PEO-pTFAP SLiC-SPE electrolyte. LFP, for which complete oxidation (deintercalation of lithium ions) can occur below 4.0 V (vs. Li/Li^+^) may thus be a suitable cathode material for examining the efficacy of PEO-pTFAP2Li as an electrolyte system, but not NCM for which a high-capacity deintercalation would require >4.0 V potential. The stability of the PEO-pTFAP2Li blend with lithium metal is uncertain. The galvanostatic cycling tests performed at 100 °C on Li|PEO-pTFAP2Li|Li cells at a current density of 0.01 mA cm^–2^ with a pulse time of 1 h showed an overpotential of ∼3 mV consistently for over 100 h, as shown in Figure S3. The voltage profile showed minor fluctuations during the test which can be attributed to the softening of the PEO matrix at elevated temperatures, making deconvolution of overpotentials difficult.

### ^7^Li NMR Line Width Analysis

**Figure 5:**
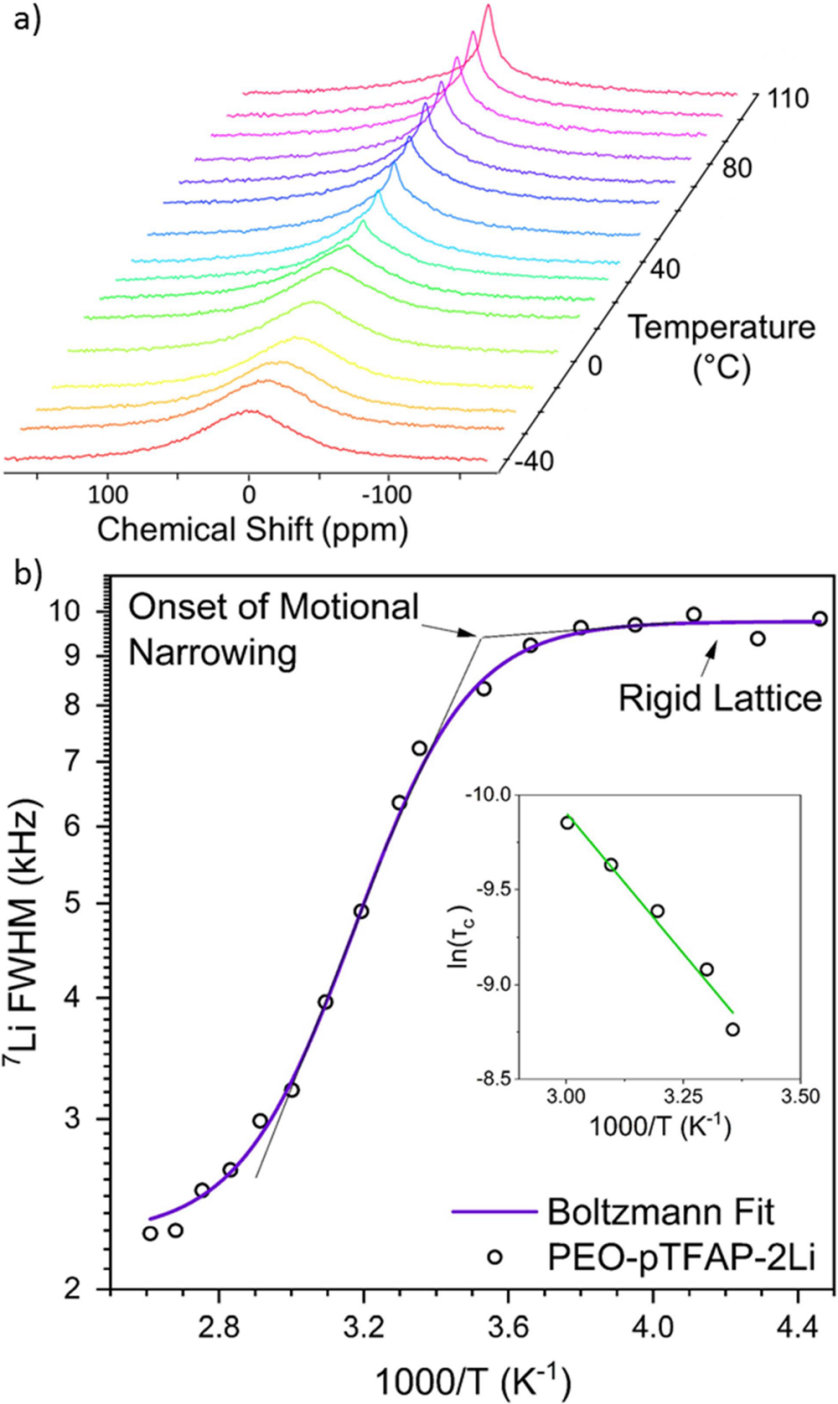
Temperature dependence of the static ^7^Li NMR spectra for a PEO-pTFAP2Li SLiC-SPE from −40 to 110 °C and (b) ^7^Li NMR linewidths plotted as a function of temperature as well as the temperature dependence of the motional correlation time demonstrating Arrhenius behavior.

Static solid-state NMR evaluations of the ^7^Li line width elucidate critical insights into the motion and mobility of Li ions in polymer electrolytes, which can be correlated to the conductivity measurements shown in Figure 2. The ^7^Li line width has a strong dependency on temperature and is shown in Figure 5a from −50 to 110 °C for the 10:1 PEO-pTFAP2Li blended electrolyte, and the full width at half-maximum (fwhm) of the peaks as a function of temperature in Figure 5b. The line width is based on a narrow component, central ^7^Li transitions (^1^/_2_ ↔ −^1^/_2_), and a broader component associated with symmetric satellite peaks due to the ^3^/_2_ ↔ ^1^/_2_ and −^1^/_2_ ↔ −^3^/_2_ quadrupolar satellite transitions.^44^ For this motional narrowing analysis, we focus on the temperature-dependent narrowing of the ^7^Li central transition because it can be directly correlated to long-range diffusion of Li-ions. The sigmoidal curve created by plotting the line width as a function of temperature can be broken down into three distinct regions (Figure 5b). At sufficiently low temperatures (below *T*_m_ of PEO in the blended electrolyte), the spectrum shows a broad and weak signal and the spectra have relatively constant fwhm; this region is known as the rigid lattice where Li-ions are essentially locked in place. With increasing temperature, the peak shape begins to rapidly narrow as Li-ions become more mobile, with the onset of motional narrowing typically being correlated to the glass transition temperature. Finally, at temperatures greater than 60 °C (PEO melts) the slope again changes to a high-temperature limit due to inhomogeneities in the magnetic field (motionally narrowed region).^45^

Further analyzing the middle-temperature region, between the onset of motional narrowing and PEO melting, the activation energy (*E*_a_) can be determined from the ^7^Li line width measurements per the Bloembergen–Purcell–Pound theory.^46^ Motional narrowing occurs when the rate of fluctuations of the local magnetic fields, known as correlation time (τ_c_), is of the order of the rigid lattice line width (Δν_0_).^47^ This relationship can be used as an estimation of the activation energy required for the motional narrowing process (lithium-ion mobility) to determine correlation times by the relation:
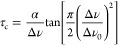
2where α is a constant and Δν is the fwhm at a given temperature. The temperature dependence of τ_c_ presents Arrhenius behavior and can be used to determine the activation energy for lithium-ion dissociation by fitting to the equation:^48^
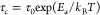
3

The activation energy of the PEO-pTFAP2Li blend in the motionally narrowed temperature range was found to be 0.26 eV, which is in good agreement with the activation energy determined by conductivity measurements (0.28 eV). Although two different temperature ranges were used for the above activation energy estimations, these data suggest that a higher temperature should facilitate the Li-ion mobility within the blend, and room temperature electrochemical performance of the cells may be limited by restricted lithium-ion mobility.

### Lithium-Ion Transference Number

**Figure 6:**
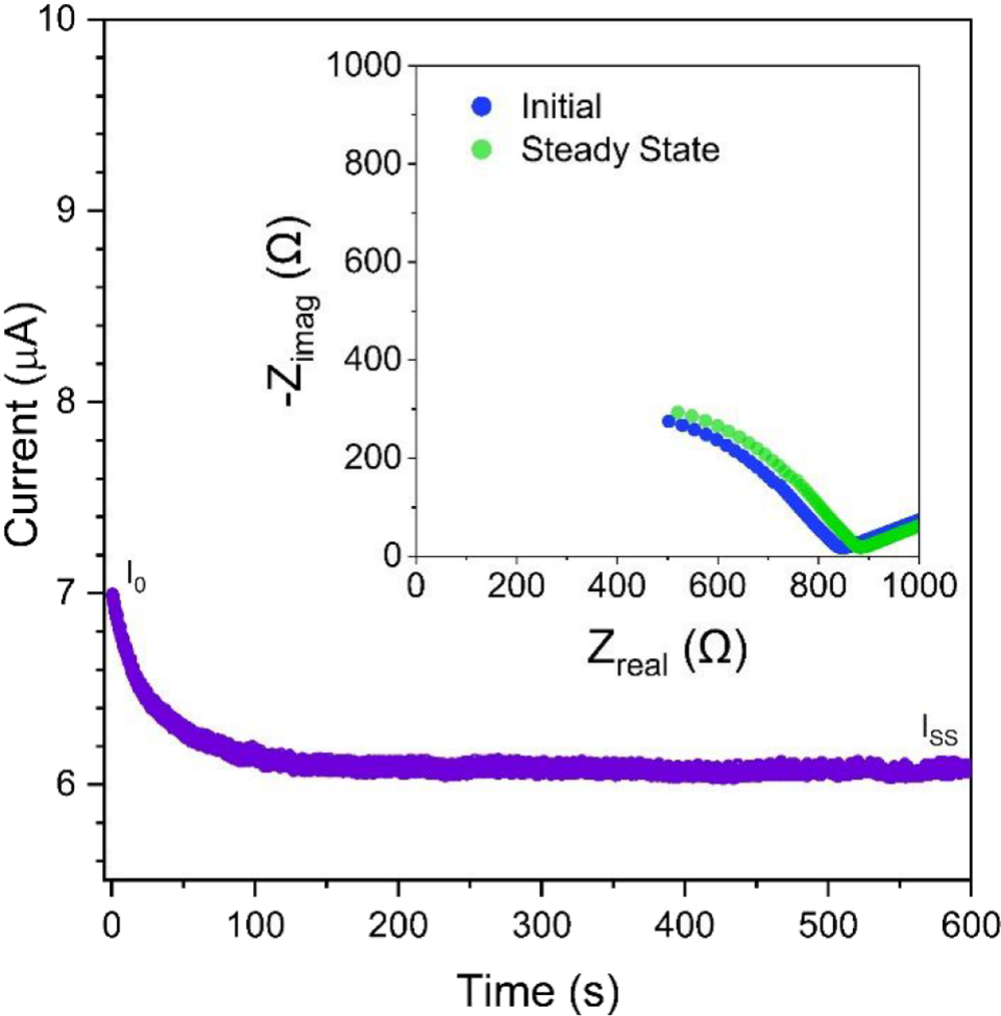
Chronoamperogram of a Li|PEO-pTFAP2Li|Li symmetric cell with a 10 mV bias voltage; *I*_0_ and *I*_SS_ represent initial and steady state currents, respectively, with Nyquist plots before and after polarization (inset).

A fundamental feature of SLiC polymer electrolytes is an immobilized anion that should lead to a Li-ion transference number (*t*_Li^+^_) close to unity. Figure 6 shows the DC polarization chronoamperogram of the PEO-pTFAP2Li SLIC-SPE in a lithium symmetric cell and the impedance spectra used for determining the Li-ion transference number (*t*_Li^+^_). From these data, the Bruce–Vincent method was used to calculate *t*_Li^+^_ = 0.76 based on the equation^49^:
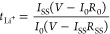
4where *I*_0_ and *I*_SS_ are the initial and steady-state current, *R*_0_ and *R*_SS_ are the initial and steady-state resistance, respectively, and *V* corresponds to the DC polarization bias (10 mV). Such *t*_Li__^+^_ value is lower than expected for any ideal SLiC-SPE (1.0) but falls within the typical range previously reported for various SLiC-SPEs.^26,50−52^ The reduced *t*_Li^+^_ value indicates some anionic movement which likely results from the PEO host matrix being in a molten state with reduced viscosity at the testing temperature (100 °C), and the applied polarization bias induces some short-range migration of the pTFAP2Li chains upon which the anions are anchored which may reduce *t*_Li^+^_. In fact, the agreement between the activation energies determined by EIS and ^7^Li NMR line width analysis implies that Li-ions are the primary contributor to the ionic conductivity of the PEO-pTFAP2Li SLiC-SPE.

### Cell Construction and Cycling Performance

**Figure 7:**
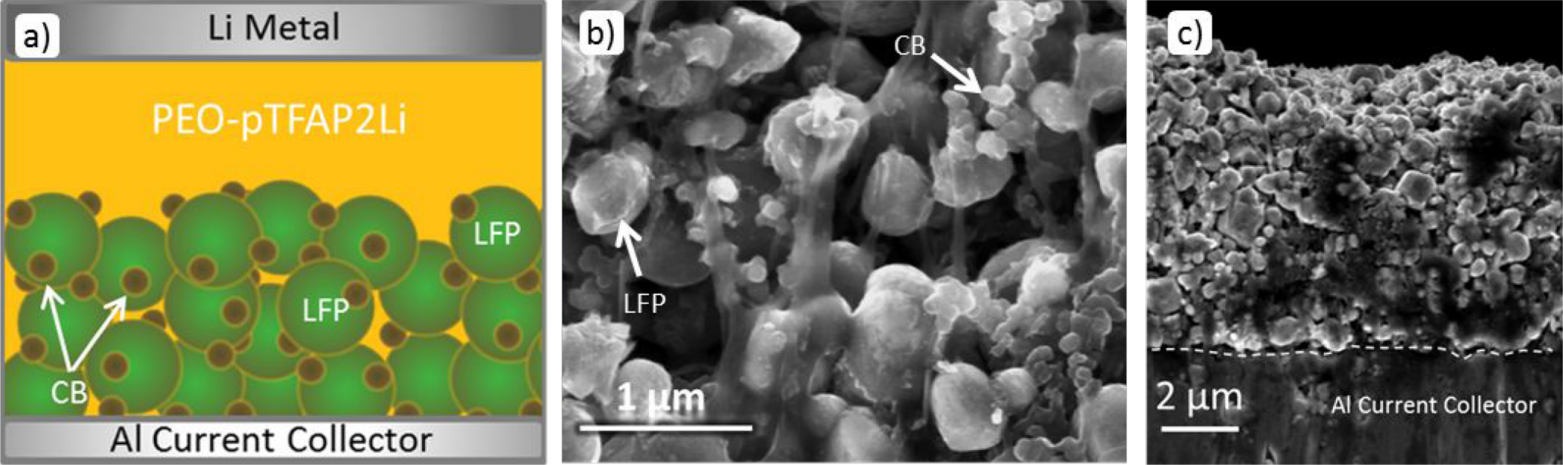
(a) Illustration of cell construction showing PEO-pTFAP2Li utilized as a binder as well as SLiC-SPE/separator promoting ionic conductivity throughout the cell, (b) FESEM micrograph of LFP cathodes, and (c) FESEM cross-section of LFP cathode with SLiC-SPE bindera.

To evaluate battery performance, the PEO-pTFAP2Li SLiC polymer electrolyte was assembled in a cell with a Li metal anode and LFP as the active cathode material (see the experimental section for details). Note that instead of using a traditional binder, PEO-pTFAP2Li served as both the cathode binder and the electrolyte (Figure 7a), which should improve the efficacy of ionic transport because commercial binders can block Li-ion transport and limit ionic conductivity within the cathode. However, this beneficial effect can be observed only if our SPE-conductive additive blend can coat the surface of all of the cathode particles to afford maximum capacity utilization. Fortunately, scanning electron microscopy (SEM) images of both the top surface and cross-section of the cathode revealed a good “wetting” between the SPE and the LFP cathode particles (Figure 7b,c).

**Figure 8:**
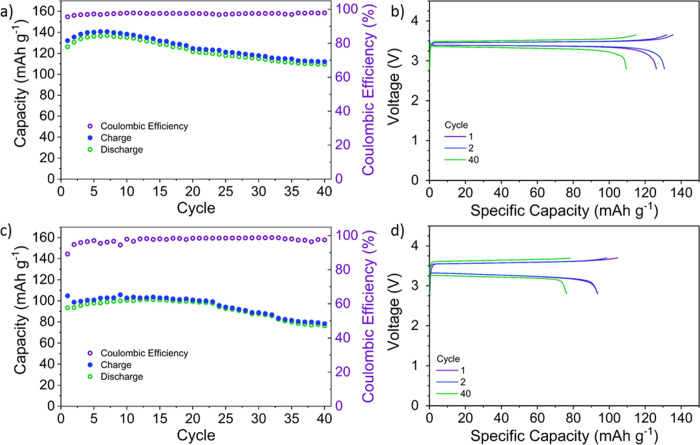
Cycling performance of the LiFePO_4_|PEO-pTFAP2Li|Li cell at (a) 0.05 and (c) 0.2 C at 100 °C and charge–discharge curves of LiFePO_4_ cathodes with PEO-pTFAP2Li SLiC-SPE at (b) 0.05 and (d) 0.2 C.

Having established this high-quality cathode fabrication, we evaluated the electrochemical performance of Li|PEO-pTFAP2Li|LFP cells at 100 °C at 0.05 and 0.2 C rates. At 0.05 C, the first cycle achieved a discharge capacity of 125 mAh g^–1^ and continued to increase for the next six cycles before reaching a maximum discharge capacity of 137 mAh g^–1^ (81% of LFP’s theoretical capacity) (Figure 8a). A slight decline in specific capacity is observed throughout the remaining cycles, with an overall 87.7% capacity retention for over 40 cycles. The cycling performance at 0.2 C exhibits similar behavior but with slightly lower values of capacities; the first cycle capacity with 93.4 mAh g^–1^ increases to a maximum of 101 mAh g^–1^ at the 14th cycle (59.4% of LFP’s theoretical capacity) with 81.8% capacity retained over 40 cycles (Figure 8b). This increase in the first few cycles and subsequent gradual decrease in specific capacity is likely related to PEO being in a molten state at the testing temperature. Initially, this creates better contact with cathode particles, and the SLIC-SPE conforms nicely to the cathode and anode surface. However, eventually, the viscous flow of PEO in combination with significant volume changes and cracking in LFP during cycling^53,54^ may lead to the gradual electrical separation of the cathode particles as the viscous binder fails to hold and electrically connect all the particles together as well as segregation of the SLiC-SPE observed during postmortem analyses shown in Figure S3. The charge–discharge (C–D) curves plotted against the potential show typical behaviors of LFP cathodes (Figure 8c,d) and a higher polarization at the higher C-rate. A gradual increase in cell polarization correlates with the observed capacity decline.

The placement of the anion near the polymer backbone reported in this work may limit the macromolecular motion, thus requiring a host polymer (such as a PEO in our study) to provide Li-ion dissociation and transport. Further improvements in the pTFAP2Li-based SPE system could come by moving the anion further down the side chain and/or replacing PEO with another polymer (or a copolymer) or a blend of polymers that is thermally active at lower temperatures, exhibits a wider ESW and retains mechanical integrity at cycling temperatures. Ultimately, the synthesis of mixed substituent SLiC polyphosphazenes through a similar chemistry with both anionic and Li-ion solvating groups (e.g., oligoether groups similar to PEO) could eliminate the need for a host polymer altogether.

## Conclusions

In summary, we report a novel synthesis route to a new class of single-ion conducting polymer electrolytes via macromolecular substitution and subsequent lithiation. Blending the lithiated polyphosphazenes with PEO allowed for the fabrication of solvent- and plasticizer-free, true all-solid-state polymer electrolytes. The 10:1 ([EO]:[Li^+^]) PEO-pTFAP2Li SLiC PE achieved an ionic conductivity of 2.1 × 10^–5^ S cm^–1^ at 100 °C, with a moderately high lithium transference number (*t*_Li^+^_ = 0.76), and is stable up to 4.0 V (vs. Li/Li^+^). Galvanostatic cycling of the fabricated PEO-pTFAP2Li blend reveals satisfactory performance with LFP cathodes achieving a maximum discharge capacity of 137 mAh g^–1^ with capacity retention of 87.7% after 40 cycles. These preliminary results of a new class of SLiC-SPEs suggest that lithiated polyphosphazenes provide a potential alternative to traditional carbon-based polymers for use in the next generation of Li metal and Li-ion batteries.

## Experimental Section

### Materials

PEO (average *M*_v_ = 600,000; Sigma-Aldrich) was dried at 50 °C for 24 h under vacuum before use. Hexachlorocyclotriphosphazene (HCCP, 99%), aluminum trichloride (AlCl_3_, 99.9%), 2-methoxyethylamine (99%), 3-methoxypropylamine (99%), *n*-butyllithium (2.5 M in hexanes), and anhydrous tetrahydrofuran (THF; 99.9%) were purchased from Sigma-Aldrich and used without further purification. 2,2,2-Trifluoroethylamine (98%), Li foil (99.9%), trimethylamine (TEA, 99%), anhydrous acetonitrile (ACN; 99.8%), and anhydrous dimethyl sulfoxide (DMSO; 99.8%) were purchased from Fisher Scientific and used as received. LiFePO_4_ (LFP, 99.5%; Gelon) and Super C45 Carbon Black were dried at 60 °C before use. All synthesis procedures were carried out on a Schlenk line under N_2_, and all other air- and/or moisture-sensitive procedures were carried out in an Ar-filled glovebox (H_2_O and O_2_ less than 1 ppm).

### Synthesis of Lithiated Polyphosphazenes

The parent polymer, polydichlorophosphzene (PDCP), was synthesized via ring opening polymerization of HCCP.^55^ HCCP (10.0 g, 28.8 mmol) and AlCl_3_ (0.30 g, 2.2 mmol) were added to a glass ampule and purged with N_2_ before flame sealing at a reduced pressure (<50 mTorr). The sealed ampule was placed in a convection oven at 260 °C for 4–6 h yielding a clear viscous gel.

Newly formed PDCP (4.2 g, 29 mmol) was dissolved in 250 mL of THF under N_2_. In a separate flask, under N_2_, a slight excess of trimethylamine (TEA; 13 mL, 92 mmol) was combined with 86 mmol of the desired amine (MEAP: 2-methoxyethylamine, MPAP: 3-methoxypropylamine, TFAP: 2,2,2-trifluoroethylamine). The TEA/amine solution was added slowly via syringe to the PDCP/THF solution in a ratio of 1:3.2:3 (PDCP:TEA:amine) and refluxed at 80 °C for 4 d to allow for complete substitution. At the end of this period, the heating was stopped and the reaction mixture was allowed to cool to room temperature. The resulting mixture was filtered to remove TEA·HCl and the filtrate was concentrated under reduced pressure using a rotary evaporator. The crude polymer was dialyzed in cellulose dialysis sacks (MW cutoff 12 kDa) first against water and then against methanol for 2 days each. The dialyzed polymer solution in methanol was concentrated using a rotary evaporator and then further dried under vacuum for 48 h to afford the dialkylamino-substituted polyphosphazenes (typical yield ∼75%), which was directly used for the lithiation.

The dialkylamino-substituted polyphosphazene (ppz) was dissolved in THF under inert conditions and cooled to −78 °C in a dry ice/acetone bath before adding *n*-butyllithium in a 1:2 (ppz:*n*-BuLi) ratio and stirred for 4 h to fully lithiate the polymer. The reaction mixture was then warmed to room temperature and the THF evaporated at a reduced pressure before transferring the lithiated polymer to an Ar-filled glovebox (H_2_O < 0.1 ppm) and washed several times with anhydrous THF to remove any impurities. Subsequently, any residual THF was removed under vacuum at 60 °C to afford the desired SLiC polymer (typical yield ∼60%).

### CBM and VBM Calculations

In this work, all density functional theory (DFT) computations were performed in VASP^56^ using Perdew–Burke–Ernzerhof XC functional^57^ and a plane-wave energy cutoff of 400 eV. The single-chain structure was utilized to model polymers, consisting of a periodic chain with two repeat units and vacuum regions (12–15 Å). The relaxed physical structures were applied to compute the electronic structure using the HSE06 functional.^58^

### Preparation of Polymer Electrolytes

SLiC polymer electrolyte membranes were prepared by blending the lithiated polyphosphazene with PEO in a predetermined [EO]:[Li^+^] ratio. Inside an Ar-filled glovebox, PEO was dissolved in ACN and the lithiated polyphosphazene in DMSO before combining the two solutions and stirring for 4 h at room temperature. The resulting viscous solution was then cast on a thin PTFE sheet secured to a flat glass plate using an adjustable height doctor blade and allowed to slowly evaporate for 2 h at room temperature before drying under vacuum at 60 °C for 24 h to remove any residual solvent.

### Li Metal Cell Fabrication

LFP cathodes were prepared inside a glovebox by dissolving 34.1 mg of PEO in 0.8 mL of ACN and separately dissolving 11.0 mg of TFAP-2Li in 0.3 mL of DMSO and combining to give 10:1 [EO]:[Li^+^]. 240 mg of LFP and 15 mg of carbon black were added to the polymer solution (80:15:5 LFP:PEO-pTFAP2Li:CB) and stirred for at least 4 h for homogeneity before casting onto aluminum foil with a doctor blade and drying on a hot plate at 80 °C for 4 h. Residual solvent was removed by further drying under a vacuum for 24 h at 80 °C. LFP mass loading ca*.* 2.1 mg cm^–2^. CR2032 cells were assembled inside the Ar glovebox with 0.75 mm Li foil as the anode.

### Electrochemical Characterization

A Gamry Interface 1000 potentiostat (Gamry Instruments, US) was used for electrochemical impedance spectroscopy (EIS), linear sweep voltammetry (LSV), and lithium transference number evaluations. The ionic conductivities of the polymer blends were evaluated via EIS in stainless steel (SS) symmetric cells (SS|PEO-ppz|SS); spectra were measured in a frequency range from 1 MHz to 0.01 Hz across a range of temperatures. At each temperature, the sample was allowed to equilibrate for 30 min before analysis. LSV was performed in a range from the open circuit voltage (OCV) to 5.5 V at 0.2 mV s^–1^ in an asymmetric cell (Li|PEO-pTFAP2Li|SS). The lithium transference number was measured using the Bruce–Vincent method,^49^ with a combination of DC polarization via chronoamperometry and EIS (before and after polarization). The Li|PEO-pTFAP2Li|Li symmetric cell was prepared inside an argon-filled glovebox before measuring the initial resistance (*R*_0_), then subjecting it to a DC bias of 10 mV to get the initial (*I*_0_) and steady state (*I*_SS_) currents and finally measuring the steady state resistance (*R*_SS_). Galvanostatic charge–discharge cycling of LFP|PEO-pTFAP2Li|Li cells at 0.05 and 0.2 C were measured on an Arbin testing system (Model No. BT2X43; Arbin Instrument, US) at 100 °C.

### Material Characterization

Solution ^1^H, ^7^Li, ^13^C, ^19^F, and ^31^P NMR spectra were acquired with a Bruker Avance III 400 MHz spectrometer and DMSO-*d*_6_ as the solvent at 298 K. For the ^31^P measurements, no internal standard was used, and the data was only used to qualitatively show a shift up/downfield to suggest a change in the local environment before and after lithiation. The phase transition behavior of PEO, pTFAP2Li, and the various blends was evaluated via differential scanning calorimetry (DSC). All experiments were performed on a Discovery DSC (TA Instruments, US), samples were hermetically sealed in aluminum pans inside an Ar-filled glovebox before analysis. During analysis, samples were first cooled to −80 °C and then heated to 150 °C at a rate of 10 °C min^–1^ under N_2_. Thermogravimetric analyses (TGA) were carried out on a Q600 thermal analyzer (TA Instruments, US) at a heating rate of 10 °C min^–1^ in an alumina crucible under N_2_. The temperature dependence of the ^7^Li NMR line width was evaluated for the 10:1 PEO:pTFAP2Li SLIC-SPE on a Bruker AVIII 400 MHz spectrometer operating at a frequency of 116.6 MHz. The sample was prepared in an Ar-filled glovebox by packing the SLIC-SPE membrane into a ZrO_2_ rotor and sealing it with a Macor cap. The sample was analyzed across a range of temperature from 223 to 383 K, allowing 30 min for equilibration before analysis. The ^7^Li chemical shifts were externally calibrated to a solid LiCl reference standard set to 0.0 ppm. The SEM imaging was performed on a SU8230 microscope (Hitachi, Japan) with an accelerating voltage of 5.0 kV and a working distance of 8 mm.

## Supporting Information Available

NMR characterization; ^1^H NMR spectra before and after lithiation, ^31^P NMR from starting material (HCCP) to parent polymer (PDCP) to amino-substituted polyphosphazene to lithiated polyphosphazene, ^13^C NMR of nonlithiated and lithiated ppzs showing no change after lithiation, and ^7^Li NMR showing a single peak suggesting complete lithiation with no Li-based impurities; ionic conductivities and activation energies of the PEO-ppz blended electrolytes at a 10:1 [EO]:[Li^+^] ratio; ionic conductivities of the PEO-pTFAP2Li blended electrolytes at all [EO]:[Li^+^] ratios; flame test for pTFAP2Li and PEO-pTFAP2Li in an ambient atmosphere; galvanostatic cycling of a Li|PEO-pTFAP2Li|Li cell tested at 100 °C; and postmortem of a LiFePO_4_|PEO-pTFAP2Li|Li cell tested at 100 °C (PDF)
