# Supplementary material for: Polyphosphazene-Based Anion-Anchored Polymer Electrolytes For All-Solid-State Lithium Metal Batteries
Source: ACS Omega. 2024 Mar 19;9(13):15410–20. doi: 10.1021/acsomega.3c10311 (PMC10993324; doi:10.1021/acsomega.3c10311)
Supplement: Supplementary file 1 — ao3c10311_si_001.pdf [file ao3c10311_si_001.pdf]

Supporting Information (SI) for

Polyphosphazene-Based Anion Anchored Polymer Electrolytes For All Solid-State Lithium Metal Batteries

Billy R. Johnson,[‡†] Ashwin Sankara Raman,[‡†] Aashray Narla,[†] Samik Jhulki,[†] Lihua Chen,[†] Seth R. Marder,[§] Rampi Ramprasad,[†] Kostia Turcheniuk,[†] and Gleb Yushin*[†]

† School of Materials Science and Engineering, Georgia Institute of Technology, Atlanta, Georgia 30332, United States

§ School of Chemistry and Biochemistry, Georgia Institute of Technology, Atlanta, Georgia 30332, United States

*Corresponding author email: yushin@gatech.edu

‡B.R.J and A.S.R. contributed equally to this work.

NMR characterization. of single lithium ion conducting (SLiC) polyphosphazenes. ^{1}H, ^{13}P, ^{13}C, and ^{7}Li NMR spectra are shown in **Figure S1.**

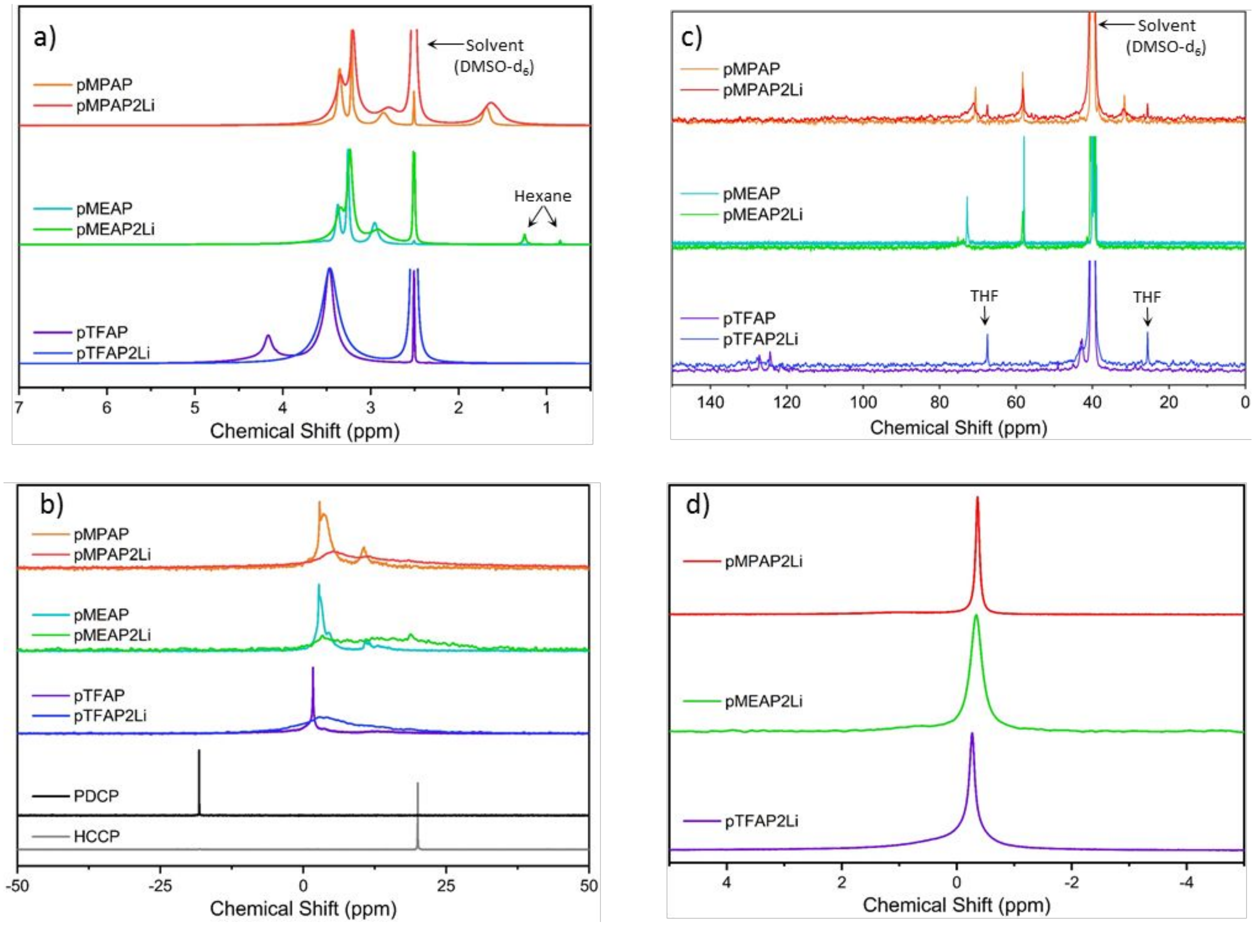

Figure S1. NMR Characterization; a) ^{1}H NMR spectra before and after lithiation, b) ^{31}P NMR from starting material (HCCP) to parent polymer (PDCP) to amino substituted polyphosphazene to lithiated polyphosphazene, c) ^{13}C NMR of non-lithiated and lithiated ppzs showing no change after lithiation, and d) ^{7}Li NMR showing a single peak suggesting complete lithiation with no Li-based impurities.

Electrochemical Characterization*.* Ionic conductivies of the 3 SLiC polyphospazenes that were synthsized along with its variation with temperature and their activation energies derived from Arrhenius plots are indicated in **Table S1**. Ionic conductivity values of PEO-TFAP-2Li blended electrolyte with different [EO]:[Li] (EO: one ethylene oxide repeat unit of PEO and two Li-ions per lithiated polyphosphazene repeat unit) ratios are indicated in **Table S2**.

Table S1. Ionic conductivities and activation energies of the PEO-ppz blended electrolytes at a 10:1 [EO]:[Li^+] ratio.

	Ionic Conductivity (σ cm^{-1})				Activation Energy (eV)	
	25 °C	40 °C	60 °C	100 °C	Before Melting	After Melting
PEO-pMEAP2Li	7.07×10^{-9}	3.73×10^{-8}	1.60×10^{-6}	6.87×10^{-6}	1.01	0.38
PEO-pMPAP2Li	3.30×10^{-8}	2.65×10^{-8}	8.30×10^{-7}	3.12×10^{-6}	1.12	0.36
PEO-pTFAP2Li	4.66×10^{-8}	3.67×10^{-7}	7.41×10^{-6}	2.11×10^{-5}	1.03	0.28

Table S2. Ionic conductivities of the PEO-pTFAP2Li blended electrolytes at all [EO]:[Li^+] ratios.

	Ionic Conductivity (σ cm^{-1})						
[EO]:[Li^+]	25 °C	40 °C	50 °C	60 °C	70 °C	80 °C	100 °C
20:1	7.82×10^{-9}	1.01×10^{-7}	3.98×10^{-7}	3.27×10^{-6}	3.95×10^{-6}	5.84×10^{-6}	8.39×10^{-6}
15:1	2.99×10^{-8}	2.34×10^{-7}	7.02×10^{-7}	4.87×10^{-6}	6.68×10^{-6}	9.04×10^{-6}	1.61×10^{-5}
10:1	4.66×10^{-8}	3.67×10^{-7}	1.02×10^{-7}	7.41×10^{-6}	1.03×10^{-5}	1.37×10^{-5}	2.11×10^{-5}
5:1	4.09×10^{-8}	1.35×10^{-8}	4.09×10^{-8}	4.15×10^{-7}	6.41×10^{-7}	9.37×10^{-7}	1.68×10^{-6}

Flame test for TFAP-2Li and PEO-TFAP-2Li blend. The flame test conducted for the TFAP-2Li shows excellent flame resistance when subjected to a propane torch at ambient atmosphere as shown in **Figure S2**. The PEO-TFAP-2Li blend showed combustion in the initial 4 seconds which subsided and remained in a charred state upto 20 s. the combustion can hence be attributed to the host polymer used to prepare the blend electrolyte.

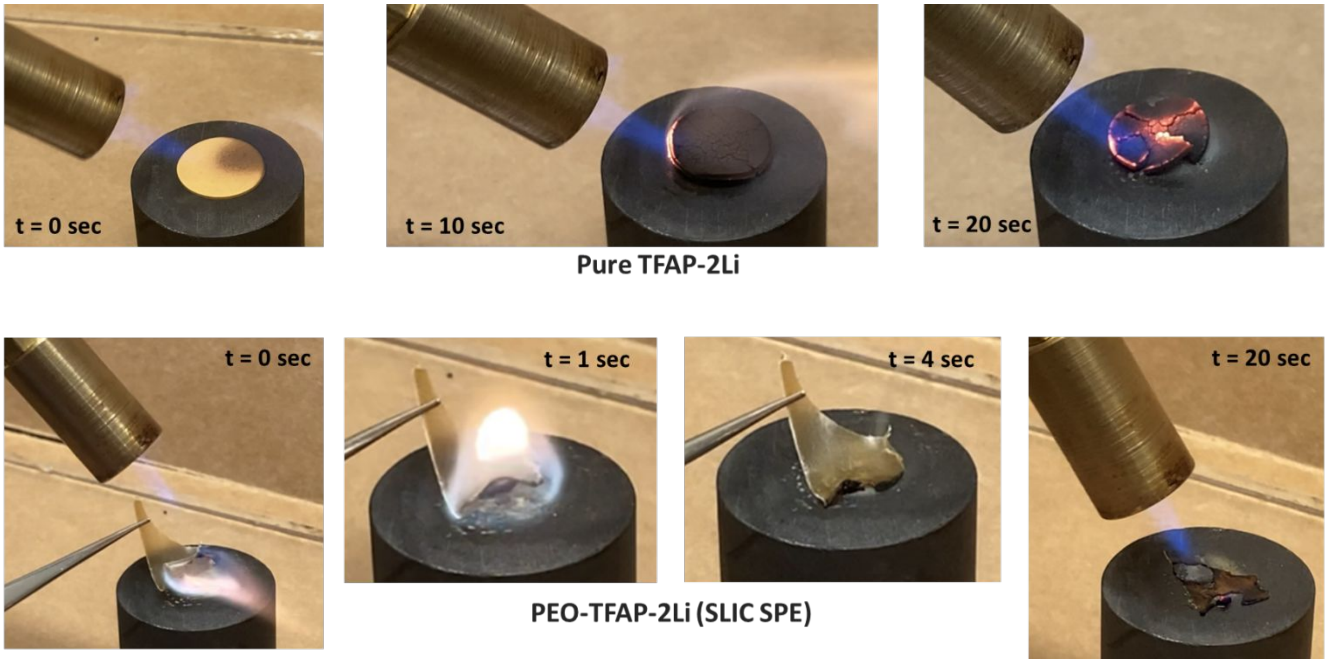

Figure S2. Flame test for pure TFAP-2Li and PEO-TFAP-2Li blend conducted using a propane torch conducted in ambient atmosphere indicating the flame resistance properties of the SLIC polyphosphazene.

Galvanostatic Cycling of Li | PEO-pTFAP2Li | Li cells. Galvanostatic cycling performance of symmetric cells made with SLiC polyphosphazene at a current density of 0.01 mA. cm^{-2} at 100°C is indicated in **Figure S3**.

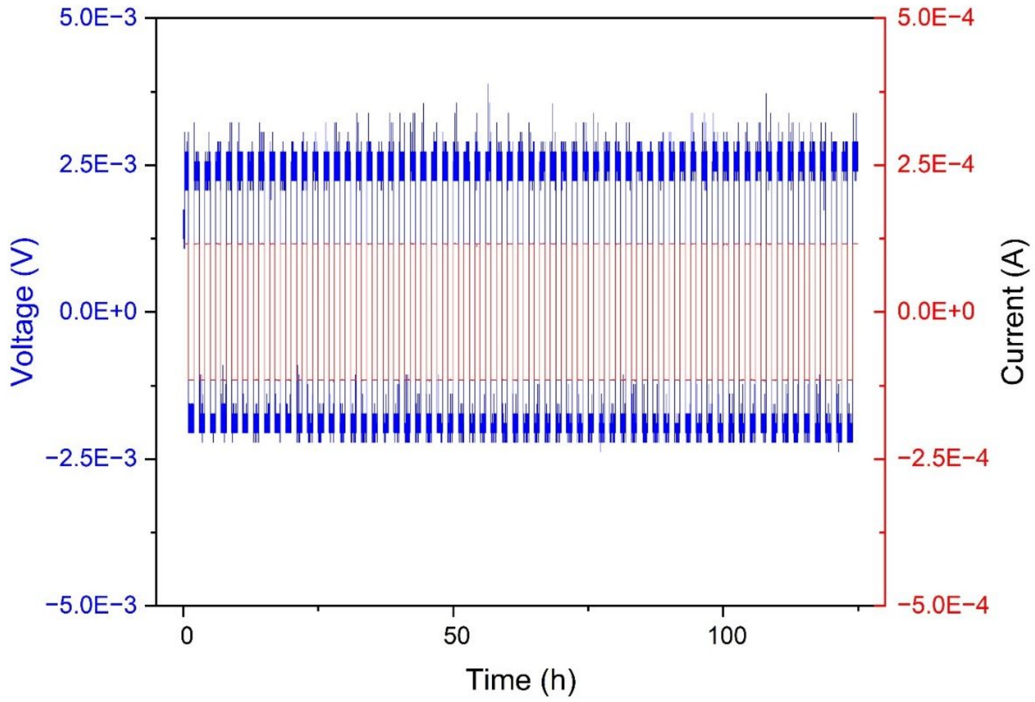

Figure S3. Galvanostatic cycling of a lithium symmetric cell with PEO-pTFAP2Li (EO:Li -10:1) electrolyte membrane at 100 °C at a current density of 0.01 mA. cm^{-2} and pulse duration of 1 h.

Postmortem Analysis. The degradation of the half cells made with SLiC polyphosphazene electrolytes blended with PEO after cycling at 0.05C at 100°C as observed visually and by using electron microscopy is shown in **Figure S4**.

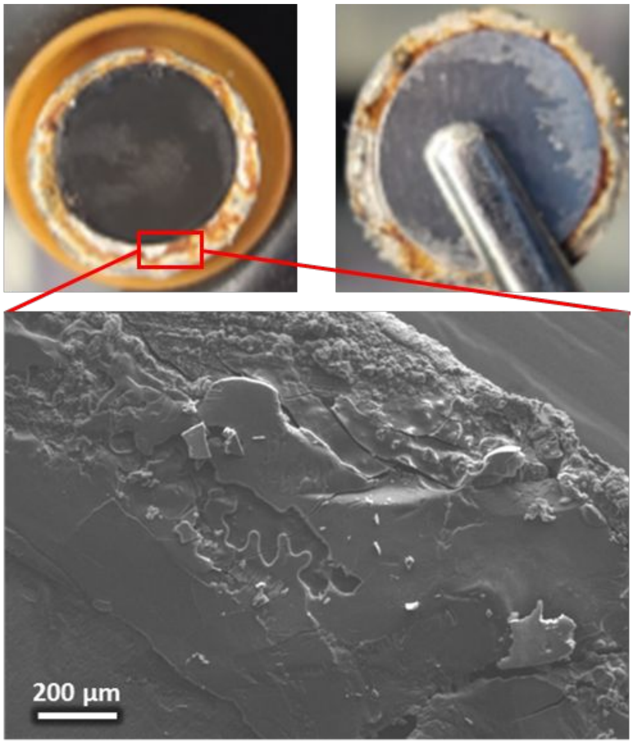

Figure S3. Post-mortem of a $LiFePO_4$ | PEO-pTFAP2Li | Li cell tested at 100 °C showing the SLiC-SPE being squeezed out slowly after cycling.
